# Should opioids be used for breathlessness and in whom? A PRO and CON debate of the evidence

**DOI:** 10.1097/SPC.0000000000000674

**Published:** 2023-10-26

**Authors:** Magnus Ekström, Daisy J.A. Janssen

**Affiliations:** a Respiratory Medicine, Allergology and Palliative Medicine, Department of Clinical Sciences Lund, Lund University, Lund, Sweden; b Department of Research and Development, Ciro, Horn; c Department of Health Services Research and Department of Family Medicine, Care and Public Health Research Institute, Faculty of Health Medicine and Life Sciences, Maastricht University, Maastricht, The Netherlands

**Keywords:** adverse events, benefit, dyspnea, morphine, palliative care

## Abstract

**Purpose of review:**

The net clinical benefit of opioids for chronic breathlessness has been challenged by recent randomized clinical trials. The purpose was to review and weigh the evidence for and against opioid treatment for chronic breathlessness in people with serious disease.

**Recent findings:**

Evidence to date on the efficacy and safety of opioids for chronic breathlessness was reviewed. Findings supporting a benefit from opioids in meta-analyses of earlier, mostly smaller trials were not confirmed by recent larger trials. Evidence pertains mostly to people with chronic obstructive pulmonary disease but also to people with pulmonary fibrosis, heart failure, and advanced cancer. Taken together, there is no consistent evidence to generally recommend opioids for severe breathlessness or to identify people who are more likely to benefit. Opioid treatment may be tested in patients with intractable breathlessness and limited other treatment options, such as in end-of-life care. Knowledge gaps were identified and recommendations were made for future research.

**Summary:**

Key Points

- Supportive findings of net benefit of opioids for chronic breathlessness in earlier trials have not been confirmed by recent larger randomized clinical trials.
- There is no evidence that the opioid treatment improves the person’s exercise capacity or quality of life, and it increases the risk of adverse events.
- Evidence to date does not support that opioids should generally be recommended for treating breathlessness.
- In people with intractable symptoms and short expected survival, with few or no treatment options, it may still be reasonable to try opioid treatment with the aim to alleviate severe breathlessness.
- Research is needed to explore the potential benefit of opioids in selected patient groups.

Opioids cannot be generally recommended for treating breathlessness based on insufficient evidence for net clinical benefit.

## INTRODUCTION

Chronic breathlessness is a common and pervasive symptom in people with serious disease^1,2^. The symptom affects daily life activities of about 10–32% of middle-aged and older people^3–5^, and especially people with cardiorespiratory diseases^6^. In palliative care, the symptom may affect the majority of people^7^ and worsen at the end of life^8^. Compared to people with pain, people with breathlessness may be less likely to be identified by clinicians as needing further evaluation, treatment, or follow-up^9^, and breathlessness can often remain ‘invisible’^10^.

Symptomatic management of chronic breathlessness often needs to be given together with curative and disease-modifying treatments, and includes identification and treatment of contributing underlying conditions, and non-pharmacological interventions such as rehabilitation, the use of a (handheld) fan^11^, walking aids such as rollator^12^, and multidisciplinary breathlessness services^13^. Pharmacological treatment with clear evidence for net clinical benefit has been lacking, but meta-analyses of mostly smaller trials suggested a beneficial effect on breathlessness of systemic (non-nebulized) opioids, such as morphine^14–16^.

However, the net benefit of opioids for chronic breathlessness was cast in doubt by recent larger randomized controlled trials (RCTs), which have shown no or conflicting effects of opioids^17–21^. Given the high burden of suffering from severe breathlessness in people with serious disease and in palliative care, discussions on how to reconcile these conflicting findings are important to inform clinical care and future research endeavors.

The aim of this narrative review is to discuss the evidence supporting (PRO) and not supporting (CON) the use of systemic opioids for treating chronic breathlessness in people with serious disease, and in which patients or settings the therapy could be considered.

The current authors also co-authored recent opioid trials^17–21^. In the present article, D.J.A.J. drafted the PRO section and M.E. the CON section, and the authors wrote the discussion together.

### Searches

Searches for relevant trials reporting on the effect of opioids for breathlessness were conducted in MEDLINE using relevant terms such as morphine/opioid and breathlessness/dyspnea/dyspnoea, from database inception through May 2023. Articles were also identified from the author’s personal reference library and from the reference lists of relevant consensus, statement and review papers in this field.

## PRO: OPIOIDS SHOULD BE USED FOR BREATHLESSNESS IN SERIOUS DISEASE

### Efficacy

#### Early trials

In the early 1980s and 1990s, smaller RCTs of opioids for breathlessness appeared. A Cochrane systematic review and meta-analysis (9 studies; 116 subjects) published in 2002^22^ further fueled the interest in this area. A cross-over trial (*n*=48) from 2003^23^ showed a potential beneficial effect of systemic (non-nebulized) opioids on breathlessness in people with serious life-limiting disease. A small to moderate, but variable, decrease in breathlessness (but no increase in exercise performance) by systemic opioids was also seen in subsequent meta-analyses in people with chronic obstructive pulmonary disease (COPD)^16^ and in people with serious (mainly cardiorespiratory) disease^14,15^. The meta-analyses included largely overlapping sets of mostly smaller trials of short duration (often single dose or up to a week’s treatment, using different oral and parental opioid substances and doses)^14–16,22^. Barnes *et al*.^14^ concluded from their Cochrane systematic review (26 studies and 526 participants) that there was low-quality evidence showing benefit of oral or parenteral opioids, while there was no evidence for the use of nebulized morphine. Although change from baseline (six studies) was not significantly different, post-treatment scores (12 studies) favored opioids compared to placebo [standardized mean difference (SMD) −0.32, 95% confidence interval (CI) −0.53 to −0.10]. A re-analysis of this meta-analysis, taking into account cross-over data, even showed a larger and clinically relevant reduction by opioids of breathlessness of 0.8 points on a 0–10 numeric rating scale (SMD −0.32, 95% CI −0.47 to −0.18)^15^.

That opioids can improve breathlessness was also supported by a laboratory-based cross-over trial of Abdallah *et al*.^24^ in 20 people with severe COPD, where oral immediate-release morphine (vs. placebo) decreased both the level of exertional ventilation and breathlessness (by a mean 1.2±0.4 Borg units) measured at a standardized time point (iso-time) during cardiopulmonary exercise testing.

#### More recent evidence in COPD

In 2020, the MORDYC study^17^, a randomized, double-blind, placebo-controlled, parallel-arm intervention study of 111 patients with COPD and moderate to very severe breathlessness, showed that regular, low-dose, oral extended-release (ER) morphine for 4 weeks was clinically relevant and statistically significant for improving disease-specific health status [COPD Assessment Test (CAT) score −2.18 points, 95% CI –4.14 to –0.22 points], which was the primary outcome of this trial. In patients with severe and very severe breathlessness [modified Medical Research Council (mMRC) breathlessness scale grades 3 and 4], the worst breathlessness improved clinically relevant and statistically significant (−1.33 points on a Numeric Rating Scale, 95% CI –2.50 to –0.16 points)^17^. Moreover, this trial showed that oral ER morphine for 4 weeks in patients with COPD is cost-effective from both the healthcare as well as the societal perspectives^25▪^.

The most recent RCT of regular, low-dose ER morphine in COPD, the BEAMS (Breathlessness, Exertion, And Morphine Sulfate) trial, including 156 participants, did not show an improvement in their primary outcome, worst breathlessness after 1 week of treatment, or in other outcomes^19▪▪^. However, an exploratory analysis of qualitative and quantitative data from this study suggested that there are so-called ‘super-responders’ among the participants^26^. These participants described the intervention as life-changing, reported easier breathing, better sleep, improved ability to walk, being able to do meaningful activities, and higher well-being. Their quantitative data showed improvement in dyspnea scores, active minutes, and active calories used, which remained after 6 months of morphine use. These findings suggest that meaningful outcomes for patients, such as perceived improvement in meaningful activities and sleep, are not reflected by the traditional outcome measures of currently available RCTs^26^. However, it should be noted that this was a secondary, exploratory analysis without a randomized comparison group.

#### More recent evidence in mixed studies or other conditions than COPD

In 2020, Currow *et al*.^18^ published a parallel-arm double-blind RCT, including 284 adults with chronic breathlessness (mMRC 2 or higher) comparing 20 mg oral ER morphine with placebo. Both groups were allowed to take as needed immediate-release morphine. COPD was the most frequent underlying condition (57%), followed by cancer (18%), mixed diseases (12%), heart failure (1%), and other illnesses. The study did not show any effect for the primary outcome of breathlessness right now. Similar to MORDYC study results in a subgroup analysis of people reporting severe breathlessness (mMRC 3 and 4), there was a small mean decrease in the secondary outcomes worst breathlessness score with morphine [mean difference on a visual analogue scale (VAS) of −7.8 mm, 95% CI −14.5 to −0.97]. Moreover, the use of rescue morphine was 55% higher in the placebo group compared to the intervention group (*P*=0.003). The same was found in an extra treatment arm including 155 people using 15 mg oral ER oxycodone for 1 week: no differences in primary outcomes, but with higher use of as needed morphine in the placebo group, which may have decreased or masked any difference between the groups^20▪▪^.

In other conditions than COPD, the available evidence is limited. A systematic review in cancer and a systematic review in heart failure did not show a beneficial effect of opioids for breathlessness ^27^,^28^. Unfortunately, studies included in these reviews often are small, of low quality, and use different opioids and routes of administration, which makes interpretation of findings difficult^27^,^28^. A study including 23 people with pulmonary arterial hypertension and a trial including 36 patients with fibrotic interstitial lung diseases (ILDs) failed to show a beneficial effect^21,29^. The latter did show that 5 mg immediate-release morphine four times daily reduced the breathlessness VAS by 11 mm (SD 3.3 mm) in the intervention group, which is clinically relevant. Nevertheless, this did not differ significantly from the placebo group [3.5 mm (SD 4.7 mm)]^29^.

Considering the challenges in studies exploring the effects of opioids on breathlessness, including the difficulties in recruitment and choosing meaningful outcome, real life data also provide relevant knowledge. In 2022, Chen and colleagues^30^ reported a prospective cohort study of all patients receiving opioid treatment [median dosage 12 (IQR 8–28) mg oral morphine equivalents a day] in an integrated respiratory and palliative care service. Among 109 patients (75% COPD; 18% pulmonary fibrosis; 2% bronchiectasis; 5% other diagnoses), 65% reported a subjective improvement in breathlessness over time, without a change in MRC score^30^.

#### Adverse events

Although meta-analysis of adverse events (AEs) could not be performed in the Ekström *et al*.^16^ systematic review due to heterogeneity of reported AEs, none of the studies reported serious AEs, no events of hypoventilation, respiratory depression, opioid-related hospitalizations, or deaths. Only temporary, treatable, and mainly mild AEs were reported such as constipation and nausea^16^. AEs in the review from Barnes *et al*.^14^ included the well-known symptoms such as constipation, nausea, vomiting, and drowsiness, while no opioid-related deaths were reported.

Verberkt and colleagues^31^ published a systematic review and meta-analysis in 2017 exploring respiratory AE of opioids for breathlessness. They included 67 studies and found no evidence for clinically relevant respiratory AE of opioids in patients with breathlessness. In fact, meta-analyses of four RCTs showed no significant decrease in arterial oxygen tension (PaO_2_), while meta-analysis of 14 RCTs showed a significant but minor decrease in arterial oxygen saturation (SaO_2_) [mean difference (MD) −0.41, 95% CI −0.71 to −0.08]. Meta-analysis of five RCTs showed a significant but clinically not relevant increase in arterial carbon dioxide tension (PaCO_2_) (MD 0.27; 95% CI 0.08–0.45). Only one single case study described a cancer patient pretreated with morphine for pain who needed temporary respiratory support following nebulized morphine for breathlessness. Non-serious respiratory depression was described in four out of 1064 patients^31^.

In the aforementioned MORDYC RCT^17^, no morphine-related hospital admissions or deaths occurred. Moreover, change in PaCO_2_ (a primary outcome of this study) did not differ significantly or clinically between the treatment groups (MD 1.2 mmHg, 95% CI −2.7 to 5.1 mmHg). Respiratory rate was lower in the morphine group (MD −1.5, 95% CI −2.8 to −0.09), without significant differences in PaO_2_, SaO_2_, SpO_2_, overnight SpO_2_, time SpO_2_ <90% during the night, and lung function parameters. AEs including constipation, nausea, vomiting, and drowsiness did not differ between the intervention and control group^17^. Also in the other studies, opioids were in general well tolerated. The common and usually transient and treatable AEs like nausea, vomiting, constipation, confusion, and drowsiness were reported in most studies^18^,^19^,^27^,^28^,^29^,^30^,^32^. In the BEAMS trial, two participants presented with respiratory failure, both in the morphine group^19▪▪^.

Finally, a population-based longitudinal cohort study in people with ILDs showed no relationship between opioids and mortality^33^, while in a population-based cohort study in COPD, dosages of ≤30 mg oral morphine equivalents a day were not associated with increased mortality in contrast to higher dosages^34^.

## CON: OPIOIDS *SHOULD NOT* BE USED FOR BREATHLESSNESS IN SERIOUS DISEASE

### Efficacy

#### Clinical trials mostly in COPD

Despite some supportive evidence in earlier, small and mostly short-term trials^15,16,22,23,14^, subsequent larger, parallel-group RCTs have not confirmed any clear effect of oral ER morphine on chronic breathlessness, compared with placebo, in people with serious disease^17^,^18^,^19^,^20^. This includes a RCT of 284 people with serious diseases (58% COPD) and moderate to severe breathlessness (mMRC ≥2), which showed no effect of 20 mg oral ER morphine over 7 days^18^. A similar lack of benefit versus placebo was seen for a separate treatment arm of 155 people with 15 mg oral ER oxycodone over 1 week^20▪▪^. The lack of benefit was seen both for the primary outcome of breathlessness and across secondary outcomes such as health-related quality of life (HrQoL). A limitation of these studies was that ‘as needed’ morphine was available to all participants and was used slightly more in the placebo groups^18^,^20^.

The MORDYC trial of 111 outpatients with COPD and chronic breathlessness (mMRC ≥2) found that oral ER morphine (twice daily, titrated to a dose of 10–30 mg/day over 4 weeks) improved self-reported health status (measured using the CAT) at 4 weeks^17^. The mean CAT total score was 2.18 (95% CI 0.16–2.50) units lower in the morphine group, which is likely to be clinically meaningful^17^. However, no clear difference in CAT scores was seen in the subgroup with more severe breathlessness (mMRC 3–4, *n*=49), which might decrease the confidence in the findings. There was no clear or consistent difference in the symptom scores between the treatment groups^17^.

In the recent BEAMS trial of 156 people with COPD and severe chronic breathlessness (mMRC 3–4), oral ER morphine titrated to 0 mg (placebo) – 32 mg over 3 weeks did not result in any difference in the intensity of ‘worst breathlessness’ at 1 week (primary endpoint), nor in any secondary efficacy outcome after 1 or 3 weeks, including no clear change in physical activity (measured mean daily step count), anxiety/depression, other symptoms, functional status, or HrQoL (including CAT)^19▪▪^. A strength of BEAMS was the measurement of physical activity (which could also be affected by morphine and confound the breathlessness assessment), and the lack of clear change in physical activity supports the validity of the findings.

#### Trials in other conditions than COPD

Lack of proven efficacy of opioids for breathlessness pertains also to other conditions than COPD. There was no evidence of benefit in a placebo-controlled cross-over trial of 20 mg ER morphine for 1 week in 23 people with pulmonary hypertension^21^, or for oral morphine 5 mg four times daily versus placebo for 1 week in 36 people with pulmonary fibrosis^29^. Recent meta-analyses have also failed to show any clear benefit of opioids for breathlessness in people with heart failure (seven RCTs; *n*=151 in intervention groups and *n*=120 in control groups)^28▪▪^, or even in people with advanced cancer (six RCTs; 107 subjects)^27^. In the latter study, types of opioids were fentanyl, hydromorphone, and morphine^27^, and there is no evidence that any type of opioid has a stronger beneficial effect on breathlessness.

### Adverse events

Opioid treatment is associated with increased risks for AEs, both when prescribed for pain^35^ and for breathlessness^19^,^28^.

A meta-analysis of RCTs and observational studies in 2017 found no evidence for serious morphine-related respiratory AEs in breathlessness trials^31^. In studies that evaluated blood gases, opioid treatment increased the partial pressure of arterial carbon dioxide (PaCO_2_) of a mean 0.27 (95% CI 0.08–0.45 kPa; five RCTs with 146 subjects) but did not affect the oxygen pressure or saturation, but there were no recorded episodes of clinically significant respiratory failure related to the treatment^31^. However, many trials were one-dose or short-term and did not assess or report AEs systematically.

Common opioid-related AEs include constipation, dry mouth, nausea, vomiting, drowsiness, confusion, tolerance, physical dependence, and withdrawal problems, which are well documented both in treatment for pain^35^ and breathlessness^14^,^17^,^18^,^19^,^21^. AEs are more likely to occur early in the treatment and at higher doses, are often transient, and respond to a decreased dose or management of the side effects, without the need to withdraw the opioid treatment. However, the tolerance to and effect of opioids varies markedly between individuals. The evaluation of AEs is complicated by the underlying disease severity and high risk of AEs in many populations.

In the MORDYC trial over 4 weeks, the risk of having one or more AEs (mostly nausea, vomiting, drowsiness, constipation, and sleepiness) did not clearly differ between the morphine and placebo groups (81% vs. 70%), nor did the rate of hospitalization (2% vs. 4%). The risk of one or more AEs was also similar in the 2020 trial (*n*=284) of morphine 20 mg versus placebo during 1 week (91% vs. 95%)^18^.

In contrast, the BEAMS trial reported a dose-dependent increased risk of AEs with morphine during the first treatment week: 48% for placebo, 64% for morphine 8 mg/day, and 78% for morphine 16 mg/day, but only few AEs resulted in treatment discontinuation^19▪▪^. There was a signal of increased serious AEs in people who had received morphine (33% vs. 12% of those with placebo), including worsening breathlessness, infections, and two events of worsening respiratory failure (both in the morphine group)^19▪▪^.

Increased risk of AEs with morphine versus placebo was also reported in a meta-analysis in people with heart failure, for nausea [29% vs. 9%; risk ratio (RR) 3.1; 95% CI 0.7–14.1], vomiting (5% vs. 1%; RR 4.3; 1.2–16.0), and constipation (32% vs. 7%; RR 4.8; 2.0–11.5)^28▪▪^.

In population studies using claims or administrative data, being prescribed opioids associates with increased all-cause mortality when prescribed for non-cancer pain^36^, and with higher risks for respiratory AEs (emergency visits and hospitalizations) and all-cause mortality in elderly people with a diagnosis of COPD^37^. In contrast, increased risks for hospitalization or death were not seen in people dispensing low-dose opioids (up to 30 mg morphine equivalent per day) in people with severe, oxygen-dependent COPD^34^ or ILD^33^. Most of the COPD and ILD patients were prescribed opioids for pain^38,39^. Of note, observational studies are prone to bias, such as confounding by the underlying disease severity, treatment indication, and differing adherence to the therapy.

## DISCUSSION

The need for optimizing palliative treatment of breathlessness is paramount. Unrelieved breathlessness has major consequences for patients and their significant others^40^. However, in many settings such as the US, (mis)use of prescription (and illicit) opioids is common and one of the leading causes of death in people 50 years or younger^35^. To limit unnecessary spread of opioids in the society, the treatment needs to be evidence-based, given at the right doses and time periods, and targeted to people who are likely to derive a net clinical benefit.

### Summary and interpretations

Taken together, the current evidence supports that:

- To date, there is no consistent scientific evidence to generally recommend use of opioids for breathlessness in people with serious disease. Opioids have not consistently been shown to improve breathlessness or HrQoL, and may increase the risk of AEs.
- There is no consistent evidence that opioids may be more beneficial in people with any specific underlying medical condition or disease severity, including in patients with COPD, pulmonary fibrosis, heart failure, or advanced cancer.
- Evidence is lacking that any opioid substance, form, or administration route, compared to another, has any better effect on breathlessness.
- However, despite the lack of consistent evidence for efficacy, the lack of effect has not been conclusively proven, and a potential benefit in some individuals has been reported.

### Implications for management

Corner stones of managing chronic breathlessness include to evaluate and optimally treat underlying medical conditions that contribute to the symptom, and non-pharmacological interventions such as mobility aids (when needed)^12^, tailored rehabilitation training, handheld fan^11^, cognitive and behavioral therapies^41^,^42^, and multidisciplinary breathlessness services^13^, to improve the subject’s ability to cope and live better with the symptom.

Evidence-based pharmacological treatment for breathlessness is currently lacking. There is no supportive evidence for benzodiazepines^43^ or antidepressants^44^. Despite the lack of consistent evidence for a benefit from opioids, there is extensive clinical experience of using opioids for severe breathlessness for people with advanced disease in palliative care and at the end of life. These may be people with intractable symptoms and short expected survival, where there are few or no treatment options. In this setting, it may still be reasonable to try opioid treatment with the aim to alleviate severe breathlessness, to selected patients without contraindications, and with close clinical follow-up and management of the dose and AE. When tested, a starting dose equivalent to 5–10 mg of oral short-acting or 10–20 mg per day ER morphine may be used for an opioid-naïve patient, and the lowest effective treatment dose should be used. At the same time, the patients should receive laxatives and close monitoring and as needed treatment of AE such as nausea. If the patient does not derive a clinical net benefit or develops an unacceptable AE, the therapy should be discontinued. The risk of AEs can be increased (including the risk of ventilatory depression) when opioids are combined with other treatments such as benzodiazepines and paroxetine^45^.

### Suggested next steps

As several larger RCTs of ER morphine in doses 0–30 mg per day in people with COPD have been published in recent years, further studies with similar design are likely not needed. A systematic review and meta-analysis of the RCTs would be valuable, including to evaluate the conflicting findings regarding opioid effects on health status (CAT)^17^,^19^. Pooling of data of existing studies might also help in learning whether there is a subgroup of patients with breathlessness who are likely to derive benefits of opioids.

Future trials should, when possible, measure breathlessness at a standardized level of exertion ^46,47^ to avoid bias from changes in physical activity [e.g. lower reported breathlessness scores in people who get less physically active due to worsening, or increased reported breathlessness in people who may improve by the treatment and become more active (which elicits more exertional breathlessness)]^47^. The proposed next research steps are listed in Table 1.

**Table 1:** Proposed next research steps regarding opioids for breathlessness

Clinically meaningful outcomes as experienced by patients and the best methods to measure these, including ecological momentary assessment (EMA)^48^
Breathlessness measured in relation to the level of exertion that was needed to elicit the symptom
New or less studied opioid substances
Treatment with short-acting vs. extended-release opioids
Biomarkers (including genetic) to identify people who are likely to derive benefits of opioids on breathlessness
Treatment effects in people with serious disease and severe breathlessness at rest

## CONCLUSION

Opioids cannot be generally recommended for treating breathlessness based on insufficient evidence for net clinical benefit.
